# Emerging threats of HPAI H5N1 clade 2.3.4.4b in swine: knowledge gaps and the imperative for a One Health approach

**DOI:** 10.3389/fvets.2025.1648878

**Published:** 2025-08-13

**Authors:** Juan Mena-Vasquez, Ana Marco-Fuertes, Marie Culhane, Montserrat Torremorell

**Affiliations:** ^1^Department of Veterinary Population Medicine, College of Veterinary Medicine, University of Minnesota, Saint Paul, MN, United States; ^2^Facultad de Veterinaria, Instituto de Ciencias Biomédicas, Universidad Cardenal Herrera-CEU, CEU Universities, Valencia, Spain

**Keywords:** HPAI, H5N1, swine, One Health, epidemiological risk, disease preparedness

## Abstract

Highly pathogenic avian influenza (HPAI) H5N1 represents a significant threat to wildlife, livestock, and public health. The recent detection of HPAI H5N1 clade 2.3.4.4b genotypes B3.13 and D1.1 in dairy cows, poultry, wild birds, wild mammals, and humans, along with the recent detection of D1.2 genotype in outdoor pigs, reflects an accelerated shift in the ecological and transmission dynamics of the virus. Given the pigs’ role in influenza ecology, these shifts present a serious threat to the swine industry and public health, accentuating the urgency for a coordinated One Health response. However, the current understanding of swine influenza, particularly in preventing and preparing for potential HPAI H5N1 incursions, has not been fully discussed. Furthermore, the consequences of such incursions on the swine industry and consequently on public health have not been explored extensively. This review addresses the knowledge gaps related to HPAI H5N1 clade 2.3.4.4b infections in pigs. Assessing the risks of HPAI H5N1 in pigs and the consequences for cross-species transmission is crucial. Preventing the introduction of HPAI into pigs and minimizing spillover risks through evidence-based strategies is vital to ensuring food security, maintaining a safe food supply, sustaining animal production systems, and preventing human infections, including potential pandemics.

## Introduction

Influenza A virus (IAV) remains a significant threat to livestock and public health due to the virus ability for interspecies transmission and reassortment, as exemplified by the swine origin 2009 H1N1 pandemic, the ongoing panzootic HPAI H5N1 outbreaks in poultry and wildlife, and the recent emergence of HPAI H5N1 clade 2.3.4.4b infections in dairy cattle (1).

Building on these historical and recent examples, concerns have intensified considerably with the emergence and panzootic spread of HPAI H5NX clade 2.3.4.4 since 2020, which has demonstrated an alarming capacity to cross species barriers (2–5).

Following its global spread, this clade was first detected in North America in 2021 (6–8), rapidly spreading and establishing among domestic poultry and in diverse wild bird species (9). Alongside its geographic spread, reassortment events with low pathogenic avian influenza (LPAI) viruses have led to the emergence of new genotypes, potentially increasing its host range and virulence (10). As evidence of this circulation, since January 2022, a total of 13,552 wild birds of different species have tested positive for HPAI H5N1 across the U.S. (as of July 11) (12), highlighting the persistence capability of this virus in its wild bird reservoir. In addition to wild birds, the U.S poultry industry has been severely affected by HPAI H5N1 (13). As of July 11 2025, approximately 174.83 million birds have been depopulated or died across all 50 states since the onset of the outbreak on February 8, 2022. A total of 1,709 flocks have been reported positive, where 788 belong to commercial and 921 to backyard operations (13).

Of particular concern, HPAI H5N1 has been detected in a wide range of mammals, including wild terrestrial and marine species, as well as farmed animals, including dairy cattle, goat, alpacas, and sheep (14–16). Most concerning, however, is the confirmation of a limited number of human cases linked to direct contact with infected animals, including poultry on farms and in live bird markets, or more recently, in dairy herds (2, 17, 18). These findings suggest an increasing adaptation of the virus to mammalian hosts and raise concerns about the virus’s zoonotic potential. Compounding this risk, several mutations associated with mammalian adaptation and neurotropism have been detected in HPAI H5N1-infected mammals, drawing significant scientific attention, as they suggest the virus’s potential evolution to efficiently infect and replicate in non-avian species (15, 19).

Consequently, marking a major host expansion, an unprecedented outbreak of HPAI H5N1 clade 2.3.4.4b genotype B3.13 was confirmed in dairy cows in Texas, U.S., on March 25, 2024 (20). This outbreak likely originated from a single spillover event from wild birds that took place in late 2023 and represents the first known incursion of HPAI H5N1 into ruminant livestock (19). This genotype rapidly expanded across the country, becoming the main genotype identified in dairy cattle to date (19). This event marked a turning point in the epidemiology of the virus and raised concerns about viral adaptation to productive mammalian species. Demonstrating continued cross-species transmission, two new independent spillovers of HPAI H5N1 clade 2.3.4.4b genotype D1.1 from wild birds were recently identified in Nevada and Arizona, on January 31, 2025, and February 13, 2025, respectively (22, 23). These events indicate that wild birds continue to serve as reservoirs for HPAI cross-species transmission, reinforcing concerns about the ongoing risk of viral introduction into livestock. As a result of these introductions, as of July 13, 2025, a total of 1,075 dairy herds have been confirmed positive in 17 U.S. states, highlighting the virus’s ability to spread beyond initial spillover events (24). These HPAI H5N1 genotypes have shown a strong tropism for the mammary tissue of lactating cows, resulting in a high viral replication within the udder (20). This leads to impaired milk production and quality, with significant economic losses to the dairy industry (25). The movement of asymptomatic and subclinically infected dairy cattle is believed to have contributed to the HPAI H5N1 herd-to-herd transmission across the U.S, complicating early detection and control efforts (19). Importantly, spillover from infected dairy cattle to poultry and dairy cattle to peridomestic species (wild or semi-domesticated animals that live near human environments) has also been documented (15, 19, 26). Affected species include grackles, blackbirds, pigeons, raccoons, and outdoor domestic cats, raising concern about the potential of secondary reservoirs in environments close to humans (15, 19, 26).

In response to growing threats of the HPAI H5N1 in dairy cattle and public health, the United States Department of Agriculture (USDA), through its Animal and Plant Health Inspection Service (APHIS), has launched three national initiatives aimed to better understand the virus epidemiology, enhance biosecurity, and strengthen occupational health protection (27). These include the National Milk Testing Strategy (NMTS) (28, 29), Interstate Movement Testing (28), and the Dairy Herd Status Program (30). Testing is conducted through diagnostic laboratories of the National Animal Health Laboratory Network (NAHLN) and the National Veterinary Services Laboratories (NVSL) (27).

Given the growing concern over cross-species transmission, including the ongoing circulation of HPAI H5N1 in dairy cattle, particular attention must be paid to swine, as this species possesses a significant susceptibility to IAVs (31). IAV is endemic in swine populations worldwide, with sustained circulation in both breeding and growing herds (32–34). This virus causes respiratory disease, contributes to indirect reproductive losses, compromises animal welfare, and represents a major economic burden to pig producers (35, 36).

The remarkable genetic and antigenic diversity of IAV is primarily due to the error-prone nature of its RNA polymerase, which results in a high mutation rate (37). Additionally, frequent reassortment events can occur when pigs are co-infected with different IAV strains, leading to the generation of novel emergent strains (37). A notable example of this dynamic is the emergence of the swine-origin H1N1pdm09 virus, which caused the first influenza pandemic of the 21st century (38). Following its spread in humans, the virus spilled back into the swine population globally, contributing to the generation and establishment of novel reassortant genotypes in pigs (31). This bidirectional exchange of viruses between pigs and humans is not rare, and it highlights the complexity of the swine-human interface. IAV from both avian and human origins contributes significantly to the introduction of new strains into swine. Avian-origin viruses are often introduced through environmental exposure or wildlife contact, whereas human-origin viruses are introduced through reverse zoonosis (39, 40). These introductions further contribute to the genetic landscape of IAVs, where multiple lineages of H1N1, H1N2, and H3N2 subtypes co-circulate in swine herds globally (31, 41–43), complicating surveillance and control efforts (44). Pigs are recognized as key intermediaries in the emergence of novel influenza strains due to their expression of both avian α2,3-Gal and human α2,6-Gal IAV receptors in their respiratory tract (45). This underscores a significant concern for the swine industry, as cross-species transmission of avian-origin and mammalian-adapted strains, along with reassortment events, may increase the risk of spillover and spread among swine, potentially leading to better-adapted strains that pose a higher risk to humans.

Although only sporadic spillover events of swine-origin variants to humans have been documented globally, these zoonotic infections have not led to sustained human-to-human transmission since the 2009 H1N1 pandemic (46, 47). Nonetheless, considering the previously reported swine-origin strains, combined with the widespread and difficult-to-control circulation of diverse IAVs in swine, the risk of future pandemics continues.

In this context, the continuous circulation of HPAI H5N1 in cattle, wild birds, and peridomestic and wild mammals (19) raises significant concerns about virus adaptation to non-avian species, including pigs, as illustrated by the recent detection of HPAI H5N1 clade 2.3.4.4b genotype D1.2 in two outdoor pigs in Oregon, U.S. (11). This highlights the need for a One Health approach, considering the interconnectivity of human, animal, and environmental health in addressing the intricate challenges posed by HPAI H5N1 infections. In light of these concerns, this review aims to synthesize current knowledge on HPAI H5N1 clade 2.3.4.4b infections in swine, identify critical knowledge gaps, and outline priorities for research that can support the swine industry in preventing the emergence and spread of novel IAVs in pigs while reducing the spillover events to humans and other animal species.

This review was part of a broader initiative commissioned by the Swine Health Information Center (SHIC) to assist the swine industry in HPAI H5N1 disease preparedness. The complete report can be found at www.swinehealth.org.

### Experimental infections and field observations of HPAI H5N1 in pigs

Given the potential impact of HPAI H5N1 on the swine industry and public health, it is crucial to understand how the virus presents in pigs. In particular, the clinical presentation and transmission of the virus within swine populations. Experimental infections provide valuable insights into how HPAI H5N1 replicates and the likelihood of transmission among pigs. Consequently, understanding the disease presentation of HPAI H5N1-infected animals is essential for effective disease preparedness.

Recently, two experimental studies investigated the pathogenesis and transmission of the bovine-derived HPAI H5N1 clade 2.3.4.4b genotype B3.13 in pigs (48, 49). In the first study, Kwon et al. (49) demonstrated that pigs are susceptible to infection with the bovine-origin HPAI H5N1 clade 2.3.4.4b genotype B3.13 virus, with replication primarily occurring in the lower respiratory tract. Despite the infection, no transmission to sentinel pigs was observed, even after direct contact. The virus caused a self-limiting infection, with limited viral shedding, and pathologic alterations were mainly localized to the lower respiratory tract. Seroconversion was observed in only two out of three principal-infected pigs at 14 days post-challenge, suggesting that pigs are moderately susceptible to this genotype (49). In contrast, Feldmann et al. (48) reported that bovine-derived HPAI H5N1 clade 2.3.4.4b genotype B3.13 caused subclinical to mild respiratory disease in pigs, with replication primarily in respiratory tissues during the first week of infection. Infectious viruses were isolated from a subset of these tissues, and shedding occurred from both the oral and nasal cavities. Pigs seroconverted, but minimal histopathologic lesions were observed. Importantly, while Kwon et al. (49) found no pig-to-pig transmission, Feldmann et al. (48) demonstrated that infected pigs could transmit the virus to a limited number of naïve sentinel pigs, as evidenced by seroconversion, although transmission remained limited. It is suggested that the timing of exposure between infected pigs and the naïve sentinels may be important, as Feldmann et al. (48) introduced the naïve pigs 24 h post-infection, while Kwon et al. (49) did so 48 h post-infection.

Together, these studies suggest that pigs are susceptible to the bovine-derived HPAI H5N1 clade 2.3.4.4b genotype B3.13; however, it is important to note that experimental conditions under which these observations were made differ substantially from field settings. Specifically, factors such as animal density, age structure, and the presence of co-infections with other endemic pathogens in commercial herds may significantly alter transmission dynamics (50). Therefore, new studies need to be conducted to support the findings regarding pathogenesis, tissue tropisms, and transmissibility, considering different age categories and co-infection in an experimental setting that allows the on-going recruitment of naïve individuals. Studies using the bovine-derived HPAI H5N1 clade 2.3.4.4b genotype D1.1, therefore, will be highly valuable.

In addition to genotype B3.13, experimental infections with other HPAI H5N1 clades have also demonstrated the susceptibility of pigs to this virus. These results revealed variable clinical presentations, lesions, and transmission risks that depend on the virus genotype and the animal species from which the virus originated (51–54). Infections with a human-derived HPAI H5N1 clade 1, a chicken-derived clade 2 (subclade 2.3), and wild birds-derived clade 2 (subclades 2.1 and 2.2) were evaluated by Lipatov et al. (54). Overall, they found that these viruses caused mild disease and limited shedding with low viral titers, and viral replication was limited to the respiratory tract. Importantly, the wild bird-derived subclade 2.2 showed similar tropism and virus titer to those of swine-origin (used as positive controls); however, the reduced number of animals limited the statistical evaluation (54). When using a chicken-derived HPAI H5N1 clade 2.3.4.4b genotype Ger-10.21-N1.5, Graaf et al. (52) observed no clinical signs, no unequivocal lesions, and limited seroconversion with marginal virus replication in one pig. In contrast, a mink-derived HPAI H5N1 clade 2.3.4.4b isolate from Spain resulted in infection, pathology of the lower respiratory tract, and 100% seroconversion but limited shedding and no transmission, concluding that pigs are highly susceptible to the mink-derived virus (53). Lastly, infections with two isolates of avian-derived (i.e., turkey and bald eagle) and two isolates of mammalian-derived HPAI H5N1 clade 2.3.4.4b (i.e., raccoon and red fox) were performed by Arruda et al. (51). Infections with avian-derived isolates showed viral replication in the lungs of pigs, resulting in approximately 50% seroconversion. In contrast, inoculation with two mammalian-derived isolates showed lung and nasal replication with 100% seroconversion and limited transmission to sentinel pigs through direct contact (51). While these findings suggest that mammalian-derived HPAI H5N1 viruses may have a slightly increased ability to infect and transmit among pigs compared to avian-origin strains, the level of transmission observed remains substantially lower than that of swine-adapted influenza viruses. Thus, caution is warranted when interpreting these results in the context of sustained pig-to-pig transmission potential.

Building upon the insights gained from experimental studies, field-based serological and virological investigations have documented avian-derived HPAI H5N1 exposure in pigs across multiple regions worldwide (Table 1). For instance, a low degree of seroconversion, indicating previous exposure to HPAI H5N1, was reported in slaughter pigs in Vietnam (55), backyard pigs in Indonesia (56), and in healthy pigs at a slaughter plant in Nigeria, where virus detection revealed HPAI H5N1 clade 2.3.2.1c (57). In 2021, HPAI H5N1 clade 2.3.4.4b seroconversion was reported in approximately 61% of free-ranging pigs co-located with poultry in Italy, despite no clinical disease reported in the pigs (58). Lastly, HPAI H5N8 clade 2.3.4.4b seroconversion was shown in backyard pigs in France (59).

**Table 1:** Field detection of various avian influenza A subtypes in pigs across different continents, countries, and years.

Continent	Country	Subtype	Clade*	Year	Clinical signs	Observations	Reference
Asia	China	H9N2		1998–2000	No		(178)
				2002	Influenza-like		(179)
				2003	Influenza-like		(150)
				2004	Influenza-like		(180)
				2013	No	Pig farm workers sampled: 6/287 (2.09%) positive	(181)
		H4N1		2009	Influenza-like	The virus is fully of avian origin	(182)
		H6N6		2010	Influenza-like	H1N1, H1N2, H9N2 and H3N2 strains were additionally detected	(183)
				2010–2012	No	Seroprevalence of H3N2 and H4N8 was also detected	(184)
		H5N1		2008–2009	No	H9N2 and H6N6 were also detected	(185)
		H10N5		2008	No	Closely homologous to the Eurasian lineage avian influenza	(186)
		H3N2		2011	Influenza-like	M gene was phylogenetically close to those in H5N1 HPAI	(187)
		H4N8		2011	Influenza-like	The NP gene was phylogenetically close to those in H5N1 HPAI	(188)
		H1N1	1C.2.3	2001–2018	No	275/293 EA H1N1 and 18/293 were EA H1N2	(189)
	South Korea	H7N2		2001	No	Genetic analysis indicates reassortment between avian H7N2 and H5N3	(190)
		H3N1		2006	Influenza-like	H3 human-like and other genes from swine influenza	(191)
		H5N2		2008	Influenza-like		(192)
	Vietnam	H5N1		2004	No	8/3125 (0.25%) tested positive	(55)
	Indonesia	H5N1	2.1.1, IDN/6/05 and 2.1.3	2005–2009	No	52/702 (7.4%) nasal swabs and 3/300 (1%) serum samples tested positive	(193)
				2006	No	10/1772 (0.56%) positive nasal swabs and 11/1786 (0.62%) positive serum samples	(56)
Europe	Belgium	H1N1		1979	Influenza-like	First supportive evidence that an influenza A virus in an avian species might have been transmitted to mammals	(194)
	England	H1N7		1992	Influenza-like	Previous H1N1 outbreak at the farm. It seems this was an equine and swine virus	(195)
	France	H5N8	2.3.4.4b	2016	No	They were all pig–poultry mixed farms	(59)
	Rome	H5N1	2.3.4.4b	2023	No	Free-ranging pigs, reared with infected poultry. Negative nasal swabs but positive seroconversion	(58)
America	Canada	H4N6		1999	Influenza-like	Phylogenetically related to waterfowl viruses. The outbreak was traced to water pumped from a lake	(119)
		H3N3		2001	Influenza-like	Phylogenetically related to waterfowl viruses. Reported on the same farm as above, so the source could be the same	(120)
		H1N1		2002	Influenza-like	Phylogenetically related to waterfowl viruses, antigenically distinct from swine influenza	(120)
	USA	N2N3		2006	Influenza-like	Isolation and characterization of H2N3 from pigs with respiratory disease from two farms in the United States and Investigation of the pathogenicity and different mammalian hosts. Transmissibility of the H2N3 isolates in	(121)
		H4N6		2015	Influenza-like	Serological analysis indicated that the virus did not efficiently transmit from pig to pig	(118)
		H5N1	Genotype D1.2	2024	Influenza-like	Shared drinking water in a mixed livestock-poultry farm in Oregon was suspected to be the source of the infection.	(11)
	Mexico	H5N2		2014-2015	Influenza-like	H1N1, H1N2 and H3N2 subtypes were also reported	(122)
Africa	Nigeria	H5N1	2.3.2.1c	2018	No	222 (44.4%) and 42 (8.4%) sera were positive for influenza A virus NP and H5 antibodies, respectively	(57)

*If known.

Considering the cumulative findings from both experimental and field studies, natural infection with HPAI H5N1 clade 2.3.4.4b genotypes B3.13 and D1.1 appears to be a very likely event. Once introduced into the swine population, these viruses may act as dead-end infections or exhibit limited transmission among pigs (48, 49, 52). However, the epidemiological risk escalates depending on the virological context of the host population. In influenza-free herds, the virus could establish more readily due to the absence of immunity and competition, potentially increasing its adaptation and facilitating onward transmission (48). Conversely, if introduced into herds already endemic for swine IAV (swIAV), co-infection could lead to genetic reassortment, raising concern over the emergence of novel viruses with altered host range, pathogenicity, or transmissibility (15, 60). These scenarios highlight the critical need to evaluate the outcomes of HPAI H5N1 introduction under both naïve and swIAV-positive scenarios.

In light of these exposure pathways and their potential implications, it becomes increasingly important to monitor for clinical outcomes that may go beyond typical respiratory signs, particularly given the broader tissue tropism reported for HPAI H5N1 clade 2.3.4.4b. While several influenza viruses, including avian, human-origin, and swine-adapted strains, are known to infect the porcine respiratory epithelium, emerging strains such as HPAI H5N1 clade 2.3.4.4b may also replicate in extra-respiratory organs, warranting closer attention to systemic manifestations. While neurologic signs have not been documented in dairy cattle during the current outbreak, this stands in contrast to observations that have been well documented globally in domestic cats (61–66), wild mammals (67–74) and farmed minks (75). Fatal systemic infections associated with neurological signs have been documented in domestic cats that consumed unpasteurized colostrum and milk from affected cows (19, 20, 26). Given this precedent, the emergence of neurological signs in pigs infected with the new bovine-derived genotypes would not be unexpected.

Extending the discussion beyond neurological manifestation, recent findings indicate that genotypes B3.13 and D1.1 have a strong tropism for the mammary gland, with milk serving as an important source of virus, raising concern about potential new routes of transmission (19, 20, 26, 76, 77). Further investigations are needed to determine whether pigs, especially lactating sows, can become infected through the mammary glands and whether this infection can spread systemically. Additionally, infections in lactating sows are needed to assess viral shedding in sows’ milk and its potential risk of infecting suckling piglets.

Beyond localized shedding routes, insights from cattle outbreaks have shown that the movement of infected animals plays a pivotal role in the interstate spread of HPAI H5N1 (19, 26). Viral RNA was more frequently detected in nasal swabs and urine in non-clinical animals (26), highlighting the role of asymptomatic transmission in herds, and also the likely contribution of other transmission routes other than milk, which could similarly drive virus spread in swine populations. Understanding the transmission dynamics of HPAI H5N1 in swine is essential to inform targeted diagnostic approaches, enhance surveillance efforts, and guide the development of effective biosecurity measures and management practices for disease prevention and control.

### Diagnostic and surveillance

Clinical signs of IAV can be easily confused with other respiratory diseases, so laboratory support is required for a definitive diagnosis (32). Due to the large and increasing diversity of IAV, it is important not only to detect but also to identify and characterize the circulating strains of IAV and to understand the limitations of current sample types and diagnostic methods (78). Selecting the right sample type and sampling method is crucial to obtain an accurate diagnosis and conduct effective surveillance in the swine population (79, 80). Respiratory tissues (e.g., lungs) and nasal swabs collected from acutely infected and febrile pigs have been historically considered the reference specimens for detecting and isolating IAVs (81, 82). However, this specimen may not be the most appropriate when seeking to detect the virus in endemically infected herds with low viral prevalence (83). Sampling of individual animals is time-consuming, and a large sample size is required to identify low-prevalence infections (84, 85). An alternative to individual pig sampling is group sampling, using specimens such as oral fluids, udder skin wipes, and nasal wipes (79, 80, 86–88). Collection of oral fluids is relatively simple and non-invasive, and for the last few years, oral fluids have become routine to surveil groups of pigs housed in pens (83). Environmental sampling using surface wipes from contaminated environments and air samples has also been used to detect IAV in swine herds and investigate pathways of virus transmission; however, their use has not been standardized, and the yield of viable isolates is, in general, poor (79, 80, 89–91). On the other hand, blood samples are suitable for detecting specific antibodies against IAV (92). The validation of the type of sample and sampling strategies will allow the implementation of better surveillance and monitoring programs for HPAI H5N1 in swine populations, particularly in the evaluation of subpopulations or production systems that are at higher risk of infection, transmission, and persistence of the virus. Additionally, these efforts will improve understanding of viral infection and immune dynamics at the individual and herd levels.

The most commonly used methods for identifying IAV include molecular techniques that detect viral genetic material, as well as sequencing and virus isolation (93). The matrix gene, which is highly conserved across IAVs, serves as the primary target for real-time reverse transcription PCR (rtRT-PCR) and enables the detection of IAVs circulating in multiple animal species (94, 95). However, RT-PCR assays targeting the hemagglutinin (HA) and neuraminidase (NA) genes are required to determine the specific virus subtype or lineage (93). Due to the extensive genetic variability of IAVs, subtyping primers are typically species- and region-specific (96). As a result, assays developed for swine-adapted IAVs may fail to detect avian-origin viruses such as HPAI H5N1. To address this issue, the World Health Organization (WHO) has published a set of protocols for the molecular identification of IAV (96). Subtype-specific RT-PCR protocols targeting HA and NA genes are also used to identify co-infections with multiple subtypes within individual pigs or pig populations (82). However, the subtype-specific protocols and commercial kits used for IAV detection in swine are not suitable for identifying specific HPAI H5N1 infections. Therefore, a specific RT-PCR assay targeting HPAI H5N1 is required for accurate diagnosis (96).

In addition to rtRT-PCR methods, conventional RT-PCR using universal primers enables amplification of all eight segments of the IAV genome, regardless of subtype, and is widely used for full-genome sequencing (97). Sanger sequencing is reliable for specific genes like HA and NA, but next-generation sequencing (NGS) is now preferred due to its higher coverage and ability to detect co-infections and minority variants (98). These methods support surveillance, virus characterization, vaccine strain selection, and molecular epidemiology (99). Sequencing can be performed directly from clinical samples (metagenomic approach), which avoids prior enrichment but may yield low IAV-specific reads due to background host material and other pathogens (100). Therefore, targeted amplification before sequencing is often preferred for accurate amplification of the IAV genome and subtype identification (101). Therefore, full-genome sequencing is recommended when HPAI H5N1 is suspected to be circulating at low levels or following potential reassortment events. Full genome sequencing remains the most effective approach to confirm viral introduction into swine populations with the subsequent identification of emerging genotypes with potential epidemiological or zoonotic implications.

While serological assays are routinely used for detecting prior exposure to swIAV, their utility for detecting infections with HPAI H5N1 is limited due to antigenic mismatch and lack of validated reagents (93). Current serological assays include hemagglutination inhibition (HI) and neuraminidase inhibition (NI) assays, which measure subtype-specific antibody responses to HA and NA proteins, respectively (93). While HI and NI assays are highly specific and useful for identifying exposure to known swine-adapted strains, they may not reliably detect antibodies generated against divergent or emerging viruses such as HPAI H5N1, especially if the reagents are not strain-matched (93). However, negative results from HI and NI assays should be interpreted with caution particularly in the context of emerging strains since false negatives can occur (93). In contrast, NP-based ELISAs offer broader reactivity across IAV subtypes but do not provide subtype-specific information (102), limiting their utility in distinguishing HPAI H5N1 from other IAVs. Therefore, the currently available serological tools for swine influenza virus surveillance may have limited sensitivity or specificity for detecting HPAI H5N1 infections. To ensure accurate serological detection of HPAI H5N1 in pigs, tailored assays using clade-specific antigens and validated for use in swine are urgently needed.

Beyond diagnostic and sampling limitations, the risk of HPAI H5N1 introduction into swine populations must also be viewed through the lens of changing ecological dynamics and cross-species transmission events. Several HPAI H5N1 spillover events have been detected between dairy cows, domestic cats, and peri-domestic species, raising growing concern over the possible establishment of new long-term reservoirs (19). Enhanced surveillance of these species, especially those close to swine production systems, is highly recommended. This is particularly relevant during colder months, when shifts in animal behavior and habitat use may increase the frequency of interspecies interaction (103). The risk is especially pronounced on areas where pigs have outdoor access or are raised in backyard or mixed-species settings.

In addition to environmental interfaces, human-mediated transmission pathways must also be considered. Agricultural fairs and exhibition shows, which are recognized as amplifiers of swIAV, could facilitate HPAI H5N1 transmission to pigs (104–106).

Determining whether HPAI H5N1 spillover into pigs results from single or multiple introduction events is critical to understanding transmission dynamics. The presence of genetically distinct viral genotypes could lead to complex epidemiological patterns, including the emergence of novel reassortants with varying clinical manifestations and pathogenicity. Moreover, the behavior of HPAI H5N1 in swine already infected with endemic swIAV or other pathogens remains largely unknown, underscoring a major knowledge gap.

These uncertainties highlight the urgent need for targeted, proactive surveillance programs for HPAI H5N1 in swine, particularly in high-risk interfaces. However, it is important to note that most influenza surveillance in swine is voluntary, and inappropriate risk communication or management could deter participation.

Overall, a more comprehensive and integrated virologic surveillance approach, spanning wild birds, poultry, livestock, peridomestic species, and humans is needed to enable timely virus characterization, improve data sharing, and support coordinated responses as the virus continues to evolve.

### Transmission routes and biosecurity

While improvements in diagnostics and surveillance are essential for early detection of HPAI H5N1 in swine, they must be complemented by a deep understanding of how the virus is introduced and spread within pig populations.

IAV can be transmitted through both direct and indirect transmission routes. Infected pigs transmit the virus through their oral and nasal secretions, as well as direct contact with susceptible pigs, representing a major transmission route (107). IAV can also be transmitted indirectly through the air and the contamination of fomites (108). When infected pigs breathe, sneeze, or cough, they release into the air a multi-disperse aerosol containing large numbers of infectious IAV particles of multiple sizes (109). These particles may remain suspended in the air or settle on surfaces, leading to environmental contamination (79, 80). Fomites, such as equipment, materials, hands, and clothing of personnel handling infected pigs, can become easily contaminated with IAV, particularly when performing farm chores. Viable IAV has been isolated from these materials, highlighting their role in indirect transmission (21). Similar patterns have been observed for HPAI H5N1, where indirect transmission via fomites, particularly through labor, shared cooperatives, and deadstock removal services, has been implicated in recent poultry and dairy outbreaks (19, 26, 110–112). Therefore, mapping these inter-farm system connections, including those involving backyard and non-commercial systems, is essential to guide monitoring and surveillance programs and prevent HPAI H5N1 incursions into the swine population.

In response to these risks, several fomite-related biosecurity measures have been implemented in the swine industry (113, 114). These measures can be related to personnel and clothing, equipment and vehicles, supplies and materials, and facility design and flow (113, 114). One example is the use of UV light boxes for decontaminating incoming supplies. Although UV-C light has been shown to inactivate IAV (115), its field efficacy against HPAI H5NX viruses remains to be validated. Additionally, the reinforcement of truck cleaning and disinfection procedures is critical, particularly given the potential for cross-contamination when using shared cattle transport systems in pigs.

The environmental stability of IAV, including avian and swine strains, is comparable under similar conditions (116, 117). According to a comprehensive review by Spackman (117), IAV can remain infectious in various substrates, including water, bedding, soil, feed, and manure, with its environmental stability varying significantly depending on temperature, pH, moisture, and matrix composition. In water, IAV can remain infectious from less than 1 day to over 80 days, with D-values ranging from 0.3 to 86.2 days depending on temperature and water chemistry. In litter and soil, virus persistence is generally limited to 3–4 days under temperate conditions (>20°C) and is reduced by factors such as heat, low pH, and porous plant-based materials like wood shavings. In feed, viable virus is rarely recovered beyond 24–48 h after contamination, with mash feed posing a higher risk than pelleted or chemically treated feed due to a lack of thermal inactivation. In manure, IAV may persist for several weeks at low temperatures (≤15°C), but is rapidly inactivated at higher temperatures (≥30°C). Composting and drying are effective in reducing viral viability, although dried manure may increase the potential for airborne dispersal of viral particles (117). However, most of this knowledge stems from experimental settings, which may not reflect real farm conditions. Prioritizing *in vivo/field* viability studies using HPAI H5N1 clade 2.3.4.4b should accurately reflect the farm environment, including multiple types of surfaces, effluents (e.g., manure, lagoon water, etc.), temperature, and relative humidity conditions. Environmental stability studies should also consider outdoor and feral pigs’ environments, which are more likely to encounter HPAI H5N1.

Evidence from sporadic infections with low pathogenic avian influenza (LPAI) viruses in pigs highlights the importance of robust biosecurity and supports targeted investigations to strengthen prevention programs (118–122). Subclinical or mild respiratory clinical signs linked to LPAI spillover have been documented, and some have adequately explained the potential source of introduction (118–122). One such case involved the probable introduction of an H4N6 subtype into a Canadian herd via water consumption from an adjacent waterfowl feces-contaminated lake (119). These findings highlight the importance of monitoring water sources and investigating water runoff for interspecies contamination. Systematic outbreak investigations are needed to identify transmission pathways and biosecurity deficiencies, aiding in designing feasible and comprehensive programs to address disease introduction through multiple routes.

The first and most recent detection of HPAI H5N1 genotype D1.2 in two backyard pigs in Oregon, U.S., reinforces this. These pigs, though asymptomatic, shared water sources, housing, and equipment with other livestock and poultry animals, and one pig was observed consuming a dead duck.

Additionally, particular attention needs to be paid in systems where feral pigs and outdoor pigs may come in contact with wild animals or extensively raised domestic animals. In the United States, more than 5 million feral swine roam across at least 35 states (123), often interacting with both wildlife and livestock in environments lacking strict biosecurity (124). Evidence of genetic reassortment and dual exposure to swine and avian IAVs in feral pigs has been reported, highlighting the potential reservoir and source of new genotypes (124–126). While direct transmission of IAV from feral to commercial swine has not been documented, the overlap between feral swine and less biosecure farms, such as backyard and farrow-to-finish operations, could facilitate viral exchange and increase the risk of introducing novel reassortant strains into domestic populations (50).

Although aerosol transmission of HPAI H5N1 in dairy remains under debate, there is no clear evidence that it plays a major role in within-herd transmission. In contrast, aerosol transmission of swIAV in pigs is well documented and poses a potential risk for both animal-to-animal and animal-to-human spread (89, 91). Even low concentrations of airborne viral particles can be epidemiologically relevant in large animal populations under favorable housing and ventilation conditions. Additionally, the deposition of airborne particles on surfaces may enhance fomite transmission, as high airborne viral loads have been associated with contamination of materials (79, 80). Given these risks, prioritizing HPAI H5N1 aerosol transmission in pigs is essential. This includes quantifying and characterizing airborne particles inside farms and at the exhaust points of ventilation systems. Monitoring air filters from swine farm ventilation systems, particularly those located near infected poultry or dairy cattle operations, could serve as an effective surveillance strategy. Supporting this approach, a field study has shown that commercial HVAC filters in swine facilities can retain detectable IAV RNA (127).

### Management practices to control the HPAI H5N1

A better understanding of the transmission dynamics of endemic swIAV has played a key role in optimizing the effectiveness of protocols to control and eliminate the virus. Such knowledge is vital in anticipating how an HPAI H5N1 incursion into pigs may present itself. The dynamics of swIAV infection in pig herds are affected by population size, immunity, movement of animals, and co-circulation of viral strains (34, 128). The infections can resolve quickly in small, closed populations (33), but in large populations, the virus can persist and maintain transmission (129, 130). Also, sequential infections of both homologous and heterologous subtypes may affect the shedding pattern and consequently increase the risk of co-infections and reassortment events (127, 131, 132). Introducing influenza-infected gilts can lead to positive pigs at weaning (129, 133, 134). Although current knowledge of swIAV transmission dynamics offers valuable insights, it is important to recognize that HPAI H5N1 viruses may exhibit distinct behavior in pigs. As discussed above, experimental and field data suggest that HPAI H5N1 infections in pigs are often subclinical and associated with limited viral replication and shedding, which may reduce transmission efficiency compared to endemic swIAV strains. Consequently, control measures may need to be adapted according to the level of adaptation and transmission potential of the HPAI H5N1 strain involved.

An effective pathway of swIAV dissemination is animal movement (84, 135, 136). To minimize the impact of HPAI H5N1 dissemination through animal movement, modeling efforts assessing other influenza viruses and means of dissemination could be extrapolated. This will enable the design of specific biocontainment strategies to prevent the spread of the virus. This information will be crucial in evaluating the effectiveness of control strategies such as prolonged gilt isolation and quarantine periods, herd closure, or vaccination in controlling and eliminating H5N1 viruses in pigs.

Immunization of sows and gilts helps reduce the prevalence of IAV-infected pigs at weaning, but it does not fully eliminate the transmission risks at weaning or in the postweaning period (131, 133, 134, 137). In addition, internal farm biosecurity practices alone have shown limited ability to prevent transmission in pre-weaned pigs (21, 137, 138). Accordingly, comprehensive influenza control programs should include practices that increase active and passive immunity and reduce viral exposure, such as gilt acclimation and vaccination, sow immunization before farrowing to enhance maternal antibody transfer, and implementation of internal biosecurity practices such as unidirectional pig flow, reduction of cross-fostering, and minimization of personnel and equipment movement between age groups. These interventions need to be validated and adjusted against HPAI H5N1 infections in pigs, depending on the risks and infection dynamics according to how well, or not, these HPAI H5N1 viruses may be adapted in the pigs.

Vaccination is the main strategy to control IAV in pigs and helps reduce clinical signs, lesions, and viral shedding (139). Vaccination may also decrease transmission, but the level of reduction depends on how well the vaccine strains induce protection against the circulating strains and also whether vaccines are used to stimulate active or passive immunity (131, 140). Vaccination of replacement animals is highly recommended, as gilt vaccination has been associated with an increased likelihood of weaning negative pigs (141). Vaccination of breeding animals, including sows before farrowing, is the most common protocol to maintain herd immunity and increase the transfer of IAV-specific antibodies to piglets (140, 142). Sow vaccination has been associated with an increased likelihood of weaning negative pigs even when vaccines do not provide perfect cross-protection (142). Seasonal vaccination and mass vaccination protocols may be used to mitigate the impact of new strain introduction into herds (141), and vaccines that limit the replication of IAV were beneficial in reducing the emergence of novel reassortant viruses under experimental conditions (143, 144).

However, vaccination does come with challenges. The continued antigenic drift, along with the high genetic diversity of IAV, complicates vaccine development (145). Therefore, effective surveillance is essential for obtaining relevant IAV isolates to ensure optimal vaccine design (141, 145). For vaccination to be effective, homologous antibodies against circulating strains must be present, as mismatched vaccine strains and field circulating strains will lead to failed virion neutralization (146). Consequently, vaccine strains need to be updated and changed frequently to confer immunity against the evolving circulating strains (147).

While current vaccination protocols are well established for endemic swIAV, their applicability to HPAI H5N1 remains uncertain. The antigenic divergence between endemic swIAV strains and HPAI H5N1 clade 2.3.4.4b viruses may limit cross-protection, necessitating the development of homologous or broadly protective vaccines. In light of the potential threat of an incursion of HPAI H5N1 in pigs, it is crucial to consider the significant delay in the production of a specific-strain vaccine in sufficient quantities for mass vaccination (148). The level of population immunity to an emerging IAV is one of the principal factors taken into account when evaluating the risk of infection (149).

One relevant component of population immunity is the presence of cross-reactive antibodies (146). Particularly, it has been demonstrated that neuraminidase inhibitory (NAI) antibodies offer extensive heterologous cross-protection against homosubtypic neuraminidases (NAs) (151). The NA is a surface glycoprotein with sialidase activity, which is crucial for releasing immature virus particles from the infected cell membrane (151). Importantly, it has been reported that pre-existing human immunity against pre-pandemic H1N1 (152, 153) and H1N1pdm09 may provide some protection against HPAI H5N1 infections due to the presence of NAI antibodies against the 2009 pandemic-origin N1 (154). This cross-protection has also been observed against the current strain circulating in dairy cattle in the U.S. (155). Prior infection with an avian-like H1N1 isolated in Belgium in 1998 partially protected pigs against a LPAI H5N1 (156). The H1N1pdm09 derived its NA protein from the avian-origin Eurasian-avian swine viruses and appears somewhat closely related to the N1 of the H5N1 virus (~84% similarity) (157). However, there is no information on the potential cross-immunity against the HPAI H5N1 generated by natural infection with currently circulating swine endemic H1N1 strains in the U.S.

In the U.S. swine population, the subtypes N1 and N2 are currently co-circulating. Specifically, the N1 lineage is predominantly represented by the “classical” swine lineage, which has been present in swine populations since the 1930s, and less defined by the pandemic N1 since 2009 (31). The endemic swIAVs are primarily managed with vaccines targeting HA and NA proteins (H1N1, H1N2, and H3N2) (158). These vaccines, whether autogenous or commercial, include whole inactivated virus vaccines (WIV), custom subunit RNA replicon particles expressing HA protein, or commercial subunit RNA replicon expressing NA proteins (44). However, the potential efficacy of these vaccines against HPAI H5N1, specifically targeting the NA protein, has not been evaluated. Therefore, it is critical to assess whether natural immunity or that induced by current commercial vaccines provides any protection against HPAI H5N1. If some level of protection exists, these vaccines could be used as an interim measure to mitigate viral incursions until a specific HPAI H5N1 vaccine becomes available. To date, no cases of HPAI H5N1 have been detected in the commercial U.S. swine population, and commercial or field-approved pig vaccines for HPAI H5N1 do not yet exist.

### HPAI H5N1 human infections and factors associated with public health concerns

HPAI H5N1 viruses can infect humans and, in some cases, cause severe disease (159). Despite the widespread circulation in animals, reported human cases remain low, with 983 sporadic infections documented since 1997, and a case fatality rate of approximately 50% (159). Since the start of the ongoing HPAI H5N1 panzootic in 2022, approximately 100 human cases have been documented across 10 countries (159). The outcome of these infections is highly variable, ranging from asymptomatic infections to deaths (159). In the U.S., 67 mild cases have been reported, primarily exhibiting symptoms of conjunctivitis and fatigue, with most cases associated with occupational exposure to cattle and poultry, one case with exposure to other animals (backyard flocks, wild birds, or other mammals), and three cases with exposure to unknown sources (160). Although the first fatal case of HPAI H5N1 in the U.S. was recently reported, there is still no evidence of person-to-person transmission (161). The limited transmission of HPAI H5N1 in humans may be due to N1-reactive antibodies (162). It is unclear whether preexisting immunity in swine workers and swine populations may protect against HPAI H5N1 infection and the emergence of novel reassortant viruses. Studies should evaluate cross-protective immunity between HPAI H5N1 clade 2.3.4.4b viruses and pandemic N1 antibodies in swine workers and pigs. Furthermore, immunity induced by HPAI H5N1 vaccines should also be assessed in pigs to enhance industry readiness against potential incursions of this virus.

The recent outbreaks of HPAI H5N1 in a mink farm in Spain (75), in fur farms in Finland (163), in marine mammals in South America (164, 165), and the most recent outbreak in dairy cows in the United States have raised concerns related to the ability of HPAI H5N1 clade 2.3.3.4b to transmit from mammal to mammal (15). Several HPAI H5N1 viruses recovered from mammalian hosts, including before the U.S. dairy cow outbreak, have harbored polymerase basic protein 2 (PB2) mutations associated with mammalian adaptation and transmissibility (4, 15). More recently, mutations in HA, matrix protein (MP), nucleoprotein (NP), PB2, and non-structural protein (NS) have been identified in HPAI H5N1 clade 2.3.4.4b genotype B3.13 from dairy cows (19), potentially contributing to increased virulence or host-range expansion (166–172). In addition, an adaptive mutation in PB2 emerged following intramammary replication of a current European HPAI H5N1 wild bird isolate (genotype euDG) (77).

In pigs, findings have been mixed. One experimental study using bovine-derived HPAI H5N1 clade 2.3.4.4b genotype B3.13 reported no detectable adaptation (48), while another experimental study using the viral genotype reported the emergence of several mutations during replication in pigs (49). Notably, most of these mutations appeared in a single pig with the highest viral titers. These included the polymerase acidic protein (PA)-I38M and NA-N307D substitutions, linked to resistance to baloxavir and oseltamivir, respectively (173, 174), as well as HA-D252Y and S136N, which are associated with enhanced binding to mammalian-like α2,6-linked sialic acid receptors (175). Importantly, classical mammalian-adaptive mutations such as PB2-E627K or D701N, and key HA mutations conferring efficient α2,6 receptor binding, were not detected (166).

Completing these findings, pigs infected with a mink-derived HPAI H5N1 clade 2.3.4.4b virus carrying the PB2-T271A, showed low-frequency mammalian-like substitutions (e.g., PB2-E627K, E627V, K526R, and HA-Q222L), though none of these variants became fixed and PB2-T271A remained stable throughout the study (53). Similarly, Arruda et al. (51) detected low-frequency substitutions across all four HPAI H5N1 clade 2.3.4.4b strains analyzed. Most substitutions occurred at functionally relevant sites, including a PB2-E627K identified in a turkey-derived virus. Additionally, polymorphisms were found in the HA gene at position 239 (R239H/C), along with receptor-binding-associated mutations (e.g., S110N, P139L, E267K, L513S) in viruses derived from red foxes and raccoons.

Should HPAI H5N1 be found in swine, it will be critical to continuously evaluate emerging mutations in the viral genome, particularly those previously associated, based on experimental or epidemiological data, with enhanced replication in mammals (mammalian adaptation), efficient transmission in pigs (pig-to-pig transmissibility), or increased human receptor binding and pathogenicity (zoonotic potential). However, functional distinctions among these effects often require *in vivo* or *in vitro* validation, as mutations may contribute to multiple phenotypes depending on context.

Surveillance of IAV in swine populations is crucial to determine the presence of the virus and the emergence of novel reassortant viruses. Factors such as the reporting of influenza-like clinical symptoms by swine workers and the use of personal protective equipment, together with vaccination and segregation of pigs, should be explored to mitigate the risk of dissemination of novel reassortant viruses should these viruses be found circulating in humans (143, 144). Furthermore, occupational exposure through aerosols is a concern since swIAV has been detected in the nares of swine workers (176, 177), and HPAI H5N1 has caused influenza-like disease in poultry and dairy workers (160). Enhanced guidance for personal protective equipment is needed, considering the variation in activities and risks across different animal species.

Finally, to address the potential emergence of a new, mammal-adapted HPAI H5N1 reassortant from infected pigs and the associated zoonotic risk, a One Health approach is essential. This requires collaboration across sectors to monitor the evolution of the virus, assess the risks of transmission, and develop preventive measures that protect both public and animal health.

## Conclusion

The expanding host range and ongoing evolution of HPAI H5N1 clade 2.3.4.4b highlight the urgent need for comprehensive surveillance, preparedness strategies, and support for scientific investigations. While experimental and field data so far suggest limited replication and transmission of HPAI H5N1 in pigs, the emergence of mammalian-adaptive mutations, even at low frequencies, demonstrates the potential for further viral evolution in this host and highlights the need for close monitoring of this virus in swine populations. As pigs serve as key hosts in the influenza virus ecosystem, they remain a critical species for the generation of reassortant viruses with pandemic potential.

Current swine influenza control strategies, including vaccination, biosecurity, and surveillance, offer a valuable foundation. However, they must be evaluated and potentially adapted to address the distinct risks posed by HPAI H5N1. In particular, cross-species transmission, mammalian adaptation, and the risk of zoonotic spillovers underscore the importance of integrating virological, epidemiological, and immunological monitoring. In addition, vaccine development strategies should explore the feasibility and efficacy of homologous and broadly protective HPAI H5N1 vaccines for use in pigs. Understanding the HPAI H5N1 immune responses in swine following natural infection or vaccination will be critical for shaping effective control programs.

Finally, mitigating the public health threat posed by HPAI H5N1 in pigs will require a One Health approach that includes coordinated efforts across veterinary, human health, and wildlife sectors to assess occupational risks, preexisting immunity in exposed populations, and to implement mitigation strategies that consider both animal and public health. The current panzootic reinforces the importance of proactive, science-based policies to prevent the emergence of the next pandemic.
